# Fusion of Near-Infrared and Raman Spectroscopy for In-Line Measurement of Component Content of Molten Polymer Blends

**DOI:** 10.3390/s19163463

**Published:** 2019-08-08

**Authors:** Shichao Zhu, Zhuoming Song, Shengyu Shi, Mengmeng Wang, Gang Jin

**Affiliations:** 1 National Engineering Research Center of Novel Equipment for Polymer Processing, South China University of Technology, Guangzhou 510640, China; 2 Key Laboratory of Polymer Processing Engineering of Ministry of Education, South China University of Technology, Guangzhou 510640, China; 3 Guangdong Provincial Key Laboratory of Technique and Equipment for Macromolecular Advanced Manufacturing, South China University of Technology, Guangzhou 510640, China

**Keywords:** data fusion, in-line spectroscopy, component content, polymer blends

## Abstract

Spectral measurement techniques, such as the near-infrared (NIR) and Raman spectroscopy, have been intensively researched. Nevertheless, even today, these techniques are still sparsely applied in industry due to their unpredictable and unstable measurements. This paper put forward two data fusion strategies (low-level and mid-level fusion) for combining the NIR and Raman spectra to generate fusion spectra or fusion characteristics in order to improve the in-line measurement precision of component content of molten polymer blends. Subsequently, the fusion value was applied to modeling. For evaluating the response of different models to data fusion strategy, partial least squares (PLS) regression, artificial neural network (ANN), and extreme learning machine (ELM) were applied to the modeling of four kinds of spectral data (NIR, Raman, low-level fused data, and mid-level fused data). A system simultaneously acquiring in-line NIR and Raman spectra was built, and the polypropylene/polystyrene (PP/PS) blends, which had different grades and covered different compounding percentages of PP, were prepared for use as a case study. The results show that data fusion strategies improve the ANN and ELM model. In particular, mid-level fusion enables the in-line measurement of component content of molten polymer blends to become more accurate and robust.

## 1. Introduction

The modified polymer generally possesses much better physical or chemical properties when compared with the virgin polymer, and therefore has found wide applications in industry. Polymer blending is one of the most commonly used polymer modification methods [1]. The properties of polymer blends are determined dominantly by the component content. Therefore, accurate and reliable technology is required for measuring the component content for ensuring the performance of the blending polymer. Nowadays, the measurement tasks are commonly accomplished off-line by methods, such as nuclear magnetic resonance [2] and thermogravimetric analysis [3]. Unfortunately, the post-processing examinations are incapable of identifying the unexpected changes of the component content timely, and the late reminder may lead to substantial scraps and pollutions. Because of these, the researchers and the polymer processing industry have paid growing attention to the in-situ measurement of the component content.

Spectroscopic techniques that are based on ultraviolet, infrared (IR), Raman, and fluorescence spectroscopy are commonly applied by analytical chemists to obtain qualitative and quantitative information of materials [4,5,6,7]. When combined with the fiber optics, these techniques allow for the on-line, in-line, or at-line measurement further. In the polymer field, there have been some reports of in-situ spectral measurement devices for polymer processing. Ingo et al. [8] developed an in-line measurement system of near-infrared (NIR), Raman, and ultrasound in polymer melt extrusion. Coates et al. [9] developed an in-process vibrational spectroscopy and ultrasound measurement system in polymer melt extrusion, and this system pump polymer melt out of the extruder, which collects spectrum in the outside channel. Jr et al. [10] developed an in-process measurement system of polymer chemical reaction in a microreactors. In real-time measurement system of polymer, spectroscopic have been widely applied in polymer synthesis [10,11] and the properties of polymer, such as particle size distribution [12], rheological properties [13], and component content [8,9,14,15,16]. However, for enriching the application of in-situ spectroscopy measurement of polymer properties (e.g., low-filled polymers), can the measuring accuracy be further improved?

Data fusion is defined as a combination of multiple sources to obtain improved information, which may improve the accuracy of the spectral analysis [17]. Generally, the data fusion methods can be categorized into three levels: low-level, intermediate-level (Mid-level), and high-level [17]. Low-level fusion is a concatenation of all the raw data, mid-level is a concatenation of extracted features of each raw data, and high-level fusion is the fusion of the results that were obtained from individual models [18]. Up to now, the data fusion has been applied to the spectral characterization in various fields. Dearing et al. [19] adopted the fused data of Raman, infrared, and nuclear magnetic resonance spectra to character the crude oil products. The result shows that the root mean square error of prediction (RMSEP) of model built by the fused data is lower than the model built by the separate spectra. Comino et al. [20] fused the data of NIR and X-ray fluorescence spectroscopy to analyze olive leaf and determine crop nutritional status. Two fusion strategy, feature fusion (Mid-level fusion) that is based on principal component scores, and measurement fusion (Low-level fusion) are applied. The prediction accuracy of nutrients is significantly promoted by applying the data fusion strategy and the feature fusion is superior to the measurement fusion.

IR spectroscopy is absorption spectrum, while Raman spectroscopy is the scattering spectrum. IR spectroscopy yields information pertaining to hydrogen bonding and asymmetric polar groups while Raman spectroscopy offers information pertaining to the molecular backbone as well as symmetrical non-polar groups [19]. The information that is contained in these two spectroscopy techniques has some complementarity. Therefore, this paper investigated the possibility to fuse the NIR spectroscopy and the Raman spectroscopy in order to find a reliable and accurate technique for in-situ measuring the component content of molten polymer blends. To achieve this aim, first, a data acquisition (DAQ) system was developed for synchronously collecting NIR and Raman spectroscopy of molten polymer during the extrusion. Subsequently, the data fusion, including the low-level fusion and the mid-level fusion, was applied for NIR and Raman spectra. Subsequently, three modelling methods, including a traditional linear model, namely the partial least squares (PLS) regression, a traditional nonlinear model, namely the artificial neural network (ANN), and a new simple and fast learning algorithm, namely the extreme learning machine (ELM) [21], were utilized for mapping the fused spectroscopic to the component content. Ultimately, the polypropylene/polystyrene (PP/PS) blends with different grades and different blending ratios were prepared for experimental verification.

## 2. Materials and Methods

### 2.1. Sample Preparation

In practical industrial production, the same polymers always have differences between grades, and it is not possible to use a single grade of plastic to prepare the blend. Thus, in this study, the used polymers were PP of three different grades and PS of two different grades, as shown in Table 1. Thirty-seven different blend samples, including 10 calibration samples and 27 prediction samples, were prepared and the details are exhibited in Table 2. These samples were firstly prepared by melt blending in a twin-screw extruder (Plasti-Corder Lab-Station, Brabender Technologie, Duisburg, Germany) at 200 °C and 150 rpm, then by air, and finally cut into pellets through a pelletizer.

### 2.2. In-Line Spectra Collection

**Figure 1:**
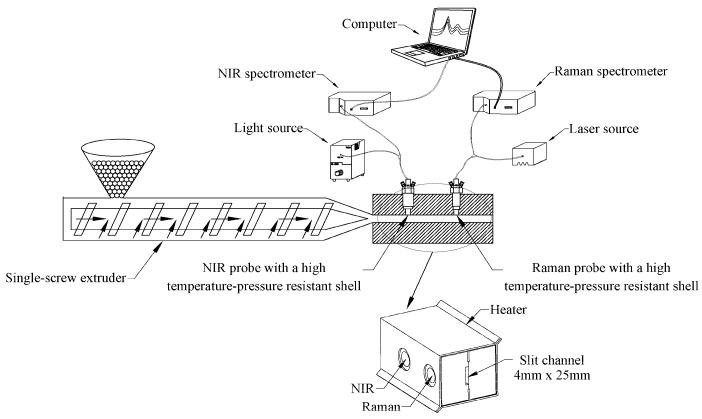
Schematic diagram of in-line near-infrared (NIR) and Raman spectroscopy measurement system.

Driven by a single-screw extruder (MESI-20, POTOP, Guangzhou, China) that was operated at a constant screw speed of 50 rpm, the PP/PS blends melts extruded through the sampling cell at a constant rate. The spectra data of PP/PS blends melts were collected in the sampling cell, which was a 25 mm width and 4 mm height slit channel at 210 °C. Figure 1 displays the measurement scheme. The in-line measurement system consisted of a sampling cell for spectral data collecting, two temperature and pressure resistant shells for protecting NIR and Raman optical probes, a Halogen source (QSPEC LS-3000, BIAOQI, Guangzhou, China), a Raman laser (Laser785-5HSB, QSPEC, Guangzhou, China) that was set at 400 mW, a NIR spectrometer (QUEST 512, Ocean Optics Inc., Orlando, FL, USA), a Raman spectrometer (QE65 Pro, Ocean Optics Inc., Orlando, FL, USA), a NIR fiber probe (QR400-7-VIS-NIR, Ocean Optics Inc., Orlando, FL, USA), a Raman fiber probe (The Raman Probe II, In Photonics, Los Angeles, CA, USA), and one computer. In-line NIR spectra of molten PP/PS blends were all collected from 1000–1600 nm, with a spectral resolution of 3.1 nm, and the Raman spectra were 1600–600 cm^−1^ with a spectral resolution of 4 cm^−1^. The NIR and Raman spectra of PP/PS molten blend of each ratio were all simultaneously collected 50 times, and the time duration of NIR and Raman for a single scan were 6 s.

### 2.3. Data Modeling

After the sampling, all of the collected data were imported into MATLAB for analysis. Pre-processes are usually required before the chemometric bi-linear modeling [22], which can increase the signal-to-noise ratio (SNR) and improve the multivariate regression model. In this paper, the baseline correction, the maximum and minimum normalization, as well as the nine-point smoothing were applied for pre-processing. Afterwards, the PLS, ANN, and ELM calibration model were built. The parameters of PLS and ELM model were optimized using cross-validation, and the number of hidden nodes in the ANN model was calculated by empirical formula as the following [23].

$$s=\log_{2}n$$

where s represents the number of hidden nodes and *n* represents the number of input data. In this case, *n* equaled 500, so the number of hidden nodes in the ANN model was 9.

### 2.4. Performance of Model

The performance of models was evaluated by the below criteria. The root mean-square error of calibration (RMSEC) is computed as:

$$RMSEC=\sqrt{\frac{\sum_{i=1}^{n}{\left(y_{i,actual}-y_{i,predicted}\right)}^{2}}{n}}$$

where $y_{i,actual}$ and $y_{i,predicted}$ represents the actual value and predicted value, respectively. Here, *n* is the sample number of calibration sets.

When it comes to the prediction set, the root mean-square error of prediction (RMSEP) is given by:

$$RMSEP=\sqrt{\frac{\sum_{i=1}^{m}{\left(y_{i,actual}-y_{i,predicted}\right)}^{2}}{m}}$$

where *m* is the number of the prediction set. Usually, the model performs better at lower RMSEC and RMSEP values.

The coefficient of determination (R^2^) is used to estimate the entire correlation between the spectral data and concentration. It is calculated via:

$$R^{2}=1-\frac{\sum_{i=1}^{m}{\left(y_{i,actual}-y_{i,predicted}\right)}^{2}}{\sum_{i=1}^{m}{\left(y_{i,actual}-\bar{y_{i,actual}}\right)}^{2}}$$

where $\bar{y_{i,actual}}$ represents the actual average value. Given the same range of ratios, the accuracy of the model will increase when R^2^ approaches 1.

The average value of each ratio’s standard deviation in the prediction results is applied to evaluate the robustness of the model, and it is calculated via:

$$\bar{S}=\frac{\sum_{i=1}^{n}S}{n}$$

where *S* represents the standard deviation, *n* represents the number of ratios, and the *S* is calculated via:

$$S=\sqrt{\frac{\sum_{i=1}^{m}{\left(y_{i,predicted}-\bar{y_{i,predicted}}\right)}^{2}}{m}}$$

where $\bar{y_{i,predicted}}$ represents the predicted average value of a single ratio and *m* is the sample number of a single ratio in the prediction sets. Usually, $\bar{S}$ reveals the undulation of data and a smaller $\bar{S}$ indicates that the model become more robust.

## 3. Result and Discussion

### 3.1. Analysis of Spectra

**Figure 2:**
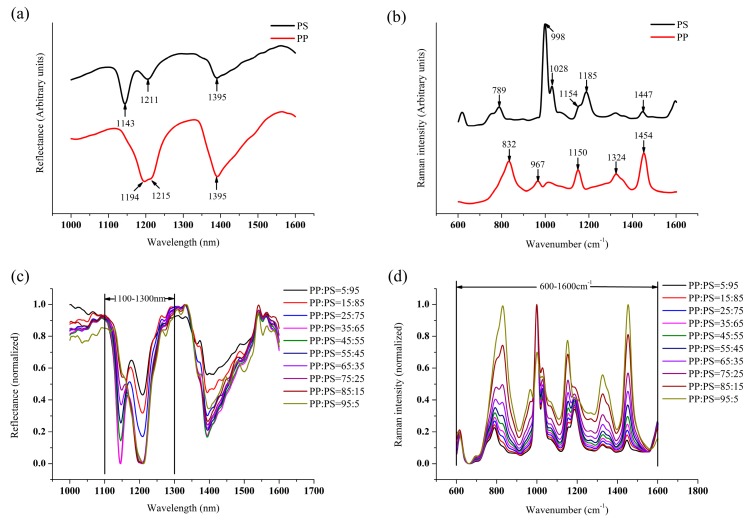
(**a**) Raw NIR spectra of polypropylene (PP) and polystyrene (PS), (**b**) Raw Raman spectra of PP and PS, (**c**) In-line NIR spectra of calibration sets, and (**d**) In-line Raman spectra of calibration sets.

**Figure 3:**
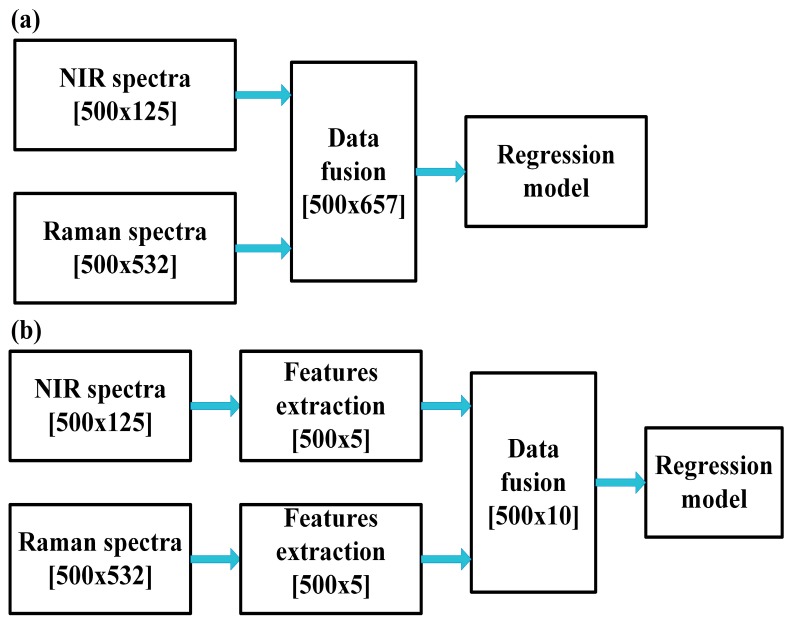
The schematic of (**a**) low-level fusion and (**b**) mid-level fusion.

The NIR and Raman spectra of virgin PP and PS melt are shown in Figure 2a,b respectively. The featured wavelength and wavenumber are marked in the figures and assigned in Table 3 [24,25]. Figure 2c,d show the in-line NIR and Raman spectra (the average of 50 spectra) after the pre-process of calibration sets. It can be seen that both the NIR and Raman spectra change regularly with the change of the blending ratio. The peak of PP decreases gradually with the decreasing PP content, and then be covered by the peak of PS. The NIR spectrum has a lot of noise despite smoothing Due to the complex environment in the extruder. Therefore, the NIR spectra ranging from 1100 nm to 1300 nm and the Raman spectra from 1600 cm^−1^ to 600 cm^−1^, which contain most of the feature information, are extracted to build the model. The extracted NIR and Raman spectra are concatenated to form the low-level fused data because the normalization process has been performed. The mid-level fused data is produced by the connection of extracted features that are gained by principal component analysis (PCA). Choosing the number of components by the high fraction of variation explained is one of the most frequently-used method [26]. In this study, the explained variance of first five principal components is 99.9%, which indicates that five principal components can represent the original data well. Therefore, five principal components are utilized to represent the spectral data. The schematic of both two fusion strategies is shown in Figure 3; 500 is the number of samples and 125 and 532 are the dimensions of NIR and Raman spectra, respectively.

### 3.2. Comparison of Models

Table 4 summarizes the validation results. Obviously, there are significant differences in the prediction results for different prediction sets, and the prediction result is worse when the grade of polymer in the prediction set is different from that in the calibration set. In the comparison of three calibration models, it can be clearly seen that the prediction results of non-linear models ANN and ELM are much more accurate than that of the PLS linear model, except the results of prediction set 3 using individual NIR spectra as the inputs, and the performance of ELM model is a little better than the ANN model. The prediction results of PLS models that are based on Raman data are very poor due to the nonlinearity of Raman spectra. 

From the viewpoint of prediction, based on the ANN and ELM models, except for the result of ELM on prediction set 3, the RMSEP is lower and R^2^ is closer to 1 when the low and mid-level fusion are applied, especially for prediction set 2 and set 3. This confirms that the data fusion strategy is satisfactorily applied, and prediction results of ANN and ELM model become more accurate. PLS linear models built by low and mid-level fused data are affected by the nonlinearity of the Raman spectra, so the prediction results of it for prediction set 3 (low-level fusion: R^2^ = 0.9607, RMSEP = 5.1196 wt. %; mid-level fusion: R^2^ = 0.9554, RMSEP = 5.4502 wt. %) show a lower degree of accuracy and higher RMSEP than the prediction results of the PLS model that was built by individual NIR spectra (R^2^ = 0.9904, RMSEP = 2.5311 wt. %). The results of the PLS models are not discussed below, due to the nonlinearity of Raman spectra.

**Figure 4:**
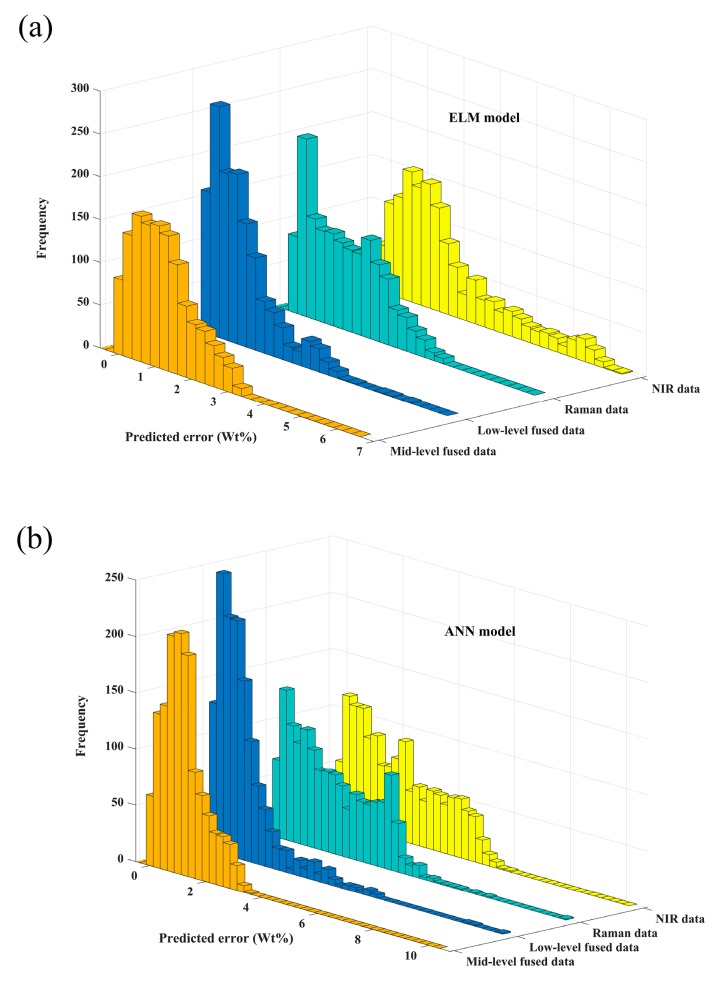
Predicted error of (**a**) extreme learning machine (ELM) and (**b**) artificial neural network (ANN) models using different data as inputs.

**Figure 5:**
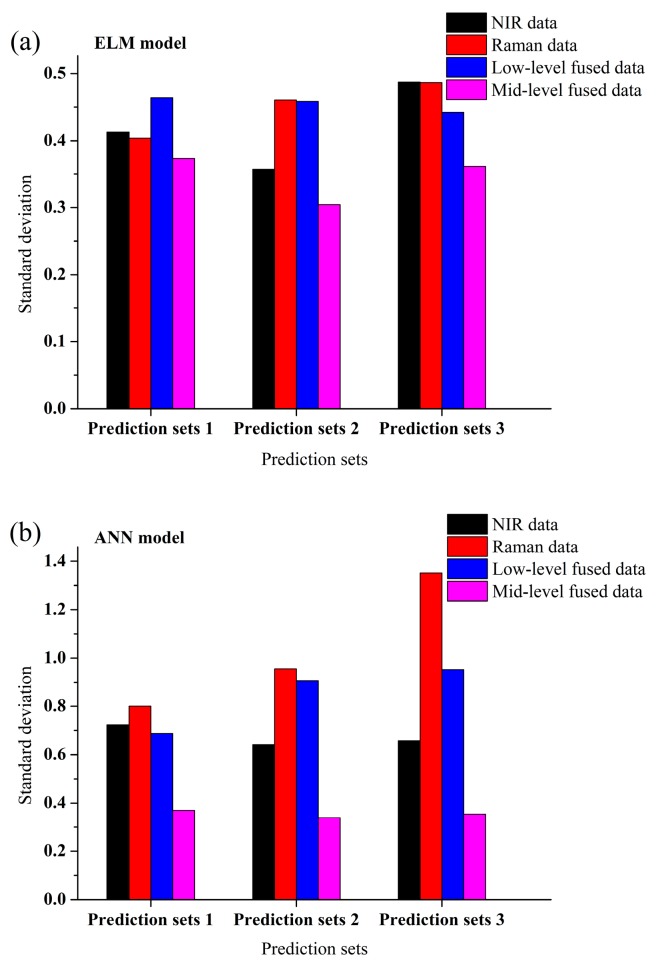
Standard deviation of (**a**) ELM and (**b**) ANN models while using different data as inputs.

**Figure 6:**
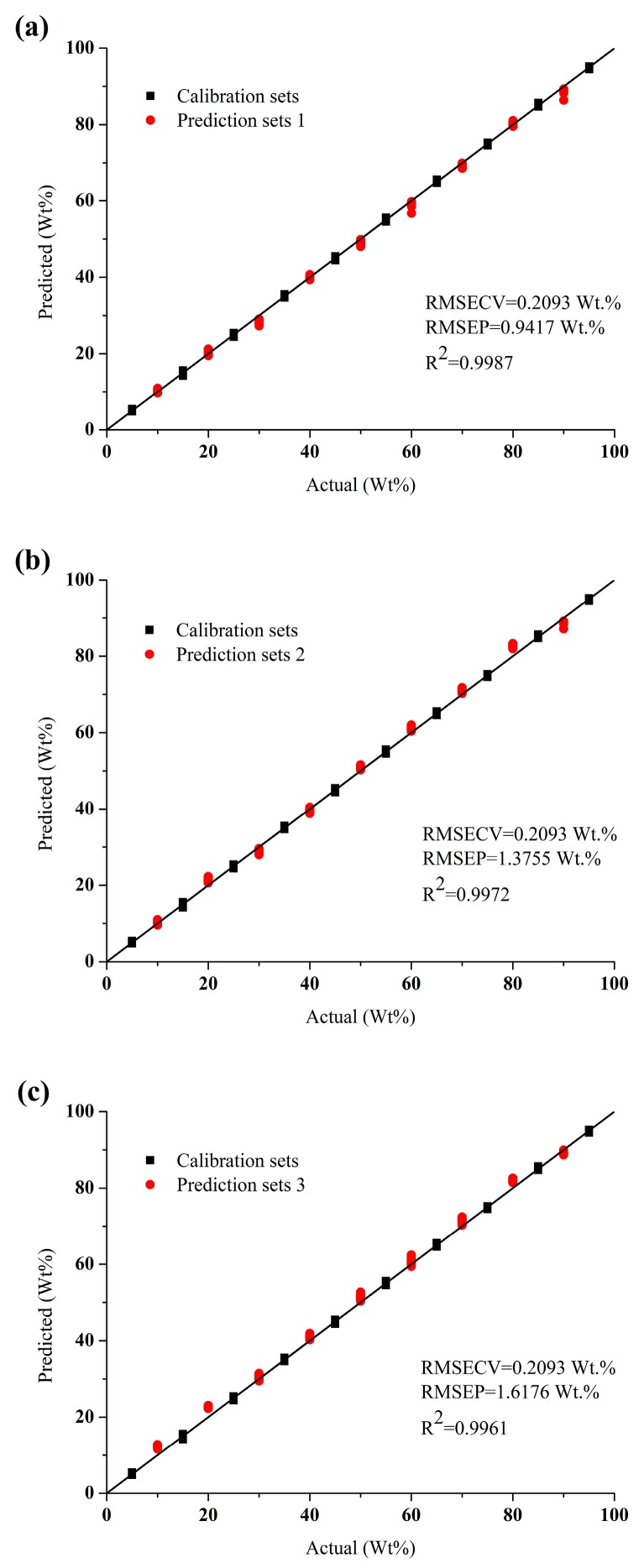
The optimal model based on calibration and (**a**) prediction set 1, (**b**) prediction set 2, (**c**) prediction set 3 using the mid-level fused data as inputs.

Figure 4 shows the predicted error distribution of ANN and ELM models while using different data as inputs in order to show the superiority of the data fusion strategy more intuitively. Whether in ANN model or ELM model, as compared with the predicted results of models built by individual spectra, the error distribution is narrower and error is smaller by applying data fusion strategies, which means that the analysis accuracy is improved. The standard deviation of ELM and ANN models using different data as inputs are shown in Figure 5. Whether in the ANN model or ELM model, as compared with the standard deviation of models built by individual spectra, the standard deviation is reduced when the mid-level fusion is applied. It is the PCA step during the mid-level fusion that reduces the undulation of data. The adoption of the mid-level fusion can further improve the robustness of model when compared with the low-level fusion. Hence, considering both accuracy and robustness, mid-level fusion is more suitable for in-line measurement of component content of molten polymer. The optimal model is the ANN model built from mid-level fused data as shown in Figure 6. The RMSEC is 0.2093 wt. % and the R^2^ and the RMSEP for prediction set 1, set 2 and set 3 are 0.9987 and 0.9417 wt. %, 0.9972 and 1.3755 wt. %, 0.9961 and 1.6176 wt. %.

## 4. Conclusions

In this study, a combination of NIR and Raman spectroscopy using data fusion strategy is researched, which aimed for an enhanced in-line measurement of component content of molten polymer blends. As for the nonlinear regression model, the benefit of the data fusion strategy in the present study is clear, because the prediction result is more accurate, especially when different grades of polymer blends are inspected.

The mid-level fusion strategy dose further improves the robustness of predictions when compared with the low-level fusion of the NIR and Raman spectroscopy. Therefore, the nonlinear regression model adopting the mid-level fused data as input can greatly improve the in-line measurement of component content of molten polymer blends while considering both accuracy and robustness. That then provides the metrological basis for process optimization and control in plastics industrial applications.

## Figures and Tables

**Table 1:** The details of materials.

Material	Grades	Density (g/cm^3^)	Melt Flow Index (g/10 min)
PP	EP548R-Basell	0.905	21
	HP741T-Basell	0.9	60
	7033N-Exxon Mobil	0.9	8
PS	158K-BASF	1.05	3.15
	PG33-QIMEI	1.05	8

**Table 2:** The details of sample set.

Sets	Grades	PP Contents
Calibration sets	PP-EP548R/PS-158K	From 95 to 5 wt. % at 10 wt. % intervals
Prediction sets 1	PP-EP548R/PS-158K	From 90 to 10 wt. % at 10 wt. % intervals
Prediction sets 2	PP-HP741T/PS-158K	From 90 to 10 wt. % at 10 wt. % intervals
Prediction sets 3	PP-7033N/PS-PG33	From 90 to 10 wt. % at 10 wt. % intervals

**Table 3:** Band assignments of in-line NIR and Raman spectra of PP and PS.

Component	NIR Band (nm)	Assignment	Raman Band (cm^−1^)	Assignment
PP	1194	Methyl C-H	1454	-CH_2_- scissoring
	1215/1395	Methylene C-H	1324	-CH_2_- twisting
			1150	C-C skeleton stretching
			967/832	-CH_3_ rocking
PS	1143	Aromatic C-H	1447	-CH- asymmetric bending
	1211/1395	Methylene C-H	1185	C-Ph asymmetric stretching
			1154/789	C-Ph asymmetric stretching
			1028	C-C asymmetric stretching in the benzene ring
			998	Benzene ring breathing

**Table 4:** Summary of model validation results.

Data Source	Sets	PLS Model		ANN Model		ELM Model	
		R^2^	RMSEP/wt.%	R^2^	RMSEP/wt.%	R^2^	RMSEP/wt.%
NIR spectra	Prediction set 1	0.9916 (11) ^a^	2.3725	0.9978 (9)	1.2211	0.9984 (99)	1.0241
	Prediction set 2	0.9858 (11)	3.0795	0.9916 (9)	2.3819	0.9928 (99)	2.1863
	Prediction set 3	0.9904 (11)	2.5311	0.9794 (9)	3.5072	0.9854 (99)	3.1249
Raman spectra	Prediction set 1	0.8335 (5)	10.5365	0.9954 (9)	1.6918	0.9986 (78)	0.9789
	Prediction set 2	0.0049 (5)	25.7559	0.9836 (9)	3.1308	0.9917 (78)	2.3571
	Prediction set 3	0.7095 (5)	13.9166	0.9929 (9)	2.2324	0.9974 (78)	1.3133
Low-level fused data	Prediction set 1	0.9963 (8)	1.6011	0.9983 (9)	1.0877	0.9994 (124)	0.6583
	Prediction set 2	0.9939 (8)	2.0200	0.9973 (9)	1.2911	0.9986 (124)	0.9507
	Prediction set 3	0.9607 (8)	5.1196	0.9946 (9)	1.8388	0.9955 (124)	1.7408
Mid-level fused data	Prediction set 1	0.9945 (9)	1.9159	0.9987 (9)	0.9417	0.9985 (47)	0.9923
	Prediction set 2	0.9909 (9)	2.4595	0.9972 (9)	1.3755	0.9970 (47)	1.4142
	Prediction set 3	0.9554 (9)	5.4502	0.9961 (9)	1.6176	0.9958 (47)	1.6803

^a^ Values in parenthesis are the number of latent variables of PLS or neurons of hidden layer.
